# Involvement of Embryo-Derived and Monocyte-Derived Intestinal Macrophages in the Pathogenesis of Inflammatory Bowel Disease and Their Prospects as Therapeutic Targets

**DOI:** 10.3390/ijms25020690

**Published:** 2024-01-05

**Authors:** Shujun Zuo, Liping Jiang, Luying Chen, Weikang Wang, Jintao Gu, Jiajie Kuai, Xuezhi Yang, Yang Ma, Chenchen Han, Wei Wei

**Affiliations:** Institute of Clinical Pharmacology, Anhui Medical University, Key Laboratory of Anti-Inflammatory and Immune Medicine, Ministry of Education, Anhui Collaborative Innovation Center of Anti-Inflammatory and Immune Medicine, Hefei 230032, China; zuoshujun2022@163.com (S.Z.); 18155235078@163.com (L.J.); cly3566064@163.com (L.C.); wangweikang1999@163.com (W.W.); 13035070790@163.com (J.G.); 2019510027@ahmu.edu.cn (J.K.); yangxuezhi@ahmu.edu.cn (X.Y.); mayang@ahmu.edu.cn (Y.M.)

**Keywords:** intestinal macrophages, inflammatory bowel disease, intestinal homeostasis, inflammatory immunity

## Abstract

Inflammatory bowel disease (IBD) is a group of intestinal inflammatory diseases characterized by chronic, recurrent, remitting, or progressive inflammation, which causes the disturbance of the homeostasis between immune cells, such as macrophages, epithelial cells, and microorganisms. Intestinal macrophages (IMs) are the largest population of macrophages in the body, and the abnormal function of IMs is an important cause of IBD. Most IMs come from the replenishment of blood monocytes, while a small part come from embryos and can self-renew. Stimulated by the intestinal inflammatory microenvironment, monocyte-derived IMs can interact with intestinal epithelial cells, intestinal fibroblasts, and intestinal flora, resulting in the increased differentiation of proinflammatory phenotypes and the decreased differentiation of anti-inflammatory phenotypes, releasing a large number of proinflammatory factors and aggravating intestinal inflammation. Based on this mechanism, inhibiting the secretion of IMs’ proinflammatory factors and enhancing the differentiation of anti-inflammatory phenotypes can help alleviate intestinal inflammation and promote tissue repair. At present, the clinical medication of IBD mainly includes 5-aminosalicylic acids (5-ASAs), glucocorticoid, immunosuppressants, and TNF-α inhibitors. The general principle of treatment is to control acute attacks, alleviate the condition, reduce recurrence, and prevent complications. Most classical IBD therapies affecting IMs function in a variety of ways, such as inhibiting the inflammatory signaling pathways and inducing IM2-type macrophage differentiation. This review explores the current understanding of the involvement of IMs in the pathogenesis of IBD and their prospects as therapeutic targets.

## 1. Introduction

Inflammatory bowel disease (IBD) is a type of intestinal inflammatory disease characterized by chronic, recurrent, remitting, or progressive inflammation, which causes the disturbance of the homeostasis between immune cells, such as macrophages, epithelial cells, and microorganisms [1]. IBD mainly includes ulcerative colitis (UC) and Crohn’s disease (CD). UC is continuous inflammation of the colon mucosa and submucosa. The disease usually involves the rectum first and then gradually spreads to the colon. CD can involve the whole digestive tract through discontinuous inflammation, and the most frequently involved sites are the end of the ileum and the colon [2]. The clinical manifestations of IBD are varied, including diarrhea, abdominal pain, blood in the stool, weight loss, etc. In severe cases, intestinal perforation, intestinal obstruction, and even cancer may occur.

Intestinal macrophages (IMs) are the first line of defense for intestinal mucosa. They have high phagocytosis and strong bactericidal properties which can resist pathogen invasion. At the same time, they help maintain the immune function of regulatory T (Treg) cells by secreting interleukin (IL)-10 and produce PGE2 to promote epithelial cell renewal. IMs play a crucial role in maintaining intestinal homeostasis [3,4]. The abnormal function of immune cells, especially IMs, is an important cause of IBD [5]. Abnormal IMs accumulate in the gut and produce a large number of proinflammatory cytokines (including TNF-α, IL-1β, IL-6, and IL-10), while recruiting and supporting T-helper 17 (Th 17) cells [6,7]. In turn, cytokines, such as granulocyte–macrophage colony-stimulating factor (GM-CSF) and IFN-γ, further enhance the proinflammatory effect of IMs, aggravating intestinal inflammation and the development of IBD [8]. IMs exist in the entire intestinal mucosa, mainly in the lamina propria, near the single-layer epithelium, and also in the smooth muscle layer of the intestinal wall, which play an important role in the recognition, phagocytosis, and degradation of cell debris and pathogens, as well as presenting antigens to T cells [9]. Macrophages present in the lamina propria, known as lamina propria macrophages (LPMs), are involved in clearing bacteria from the epithelial barrier. Macrophages exist in the smooth muscle layer of the intestinal wall, also known as muscularis macrophages (MMs), and play an important role in regulating intestinal movement. Meanwhile, part of IMs is derived from embryonic precursors that self-renew in situ (embryo-derived IMs (EIMs)), while the other part is differentiated by monocytes in blood after perforating blood vessels (monocyte-derived IMs (MIMs)) [10]. EIMs can perform in situ self-renewal, effectively clearing bacteria and apoptotic cells from the intestine. MIMs mainly include IM1 and IM2 types, which play an important role in immune regulation. After EIMs enter the intestine, the size of monocytes increases, the endoplasmic reticulum and mitochondria proliferate, lysosomes increase, and phagocytosis function is enhanced [11].

The function of IMs is affected by many factors, such as intestinal epithelial cells, intestinal fibroblasts, and intestinal flora. At the same time, IMs can also regulate their effects on the intestine in turn. IMs are able to change the inflammatory phenotype and secrete inflammatory factors to interact with intestinal epithelial cells, intestinal fibroblasts and intestinal flora, balance the possible responses to beneficial intestinal flora and pathogen stimulation, and play functions such as phagocytosis, antigen presentation, immune regulation, wound healing, and tissue repair in the process of intestinal homeostasis. They affect the remission and progression of IBD.

Currently, the clinical drugs for IBD mainly include 5-aminosalicylic acid (5-ASA), corticosteroids, immunosuppressants, and TNF-α inhibitors, which have limited efficacy and are prone to clinical adverse reactions, such as anemia, rash, and liver, as well as pancreas injury, and even lead to adverse events, such as infection, nervous system diseases, and malignant tumors. Therefore, the search for new treatments has become an urgent task. Studies have shown that regulating the polarization of IMs and inhibiting the release of proinflammatory cytokines may be new therapeutic strategies for regulating intestinal inflammation, and regulating the phenotype of IMs by inhibiting the proinflammatory M1 subgroup and/or inducing the anti-inflammatory M2 subgroup may effectively improve IBD [12,13]. Based on the important role of IMs in intestinal inflammation and remission, targeting IMs to suppress inflammation and promote mucosal healing may be a potential therapeutic target for IBD.

## 2. Physiological Functions of IMs

**Figure 1:**
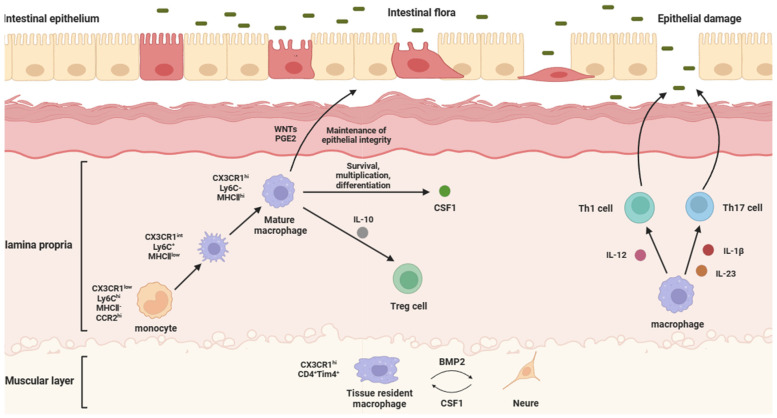
Physiological functions and intestinal effects of different IMs. The structure of the intestine can be divided into the epithelium, the lamina propria, and the muscularis, from the inside to the outside, in which intestinal macrophages are mainly distributed in the lamina propria and muscularis. Laminae propria macrophages are mostly differentiated from blood-derived monocytes. They secrete WNTs and PGE2 to maintain the integrity of the intestinal epithelial barrier, IL-10 to regulate the immune function of Treg cells, and CSF1 to maintain survival, proliferation, and differentiation. When intestinal flora erode intestinal epithelial cells, epithelial damage will be caused. At this time, laminae propria macrophages will secrete IL-12 to act on Th1 cells, while secreting IL-23 and IL-1β to act on Th17 cells, in order to activate the immune response, reduce the inflammatory response, and repair the epithelial barrier. Muscularis macrophages are principally tissue-resident macrophages, which can secrete BMP2 to support the differentiation of intestinal neurons, while intestinal neurons can secrete CSF1 to support the local maintenance of muscularis macrophages.

IMs are present in the mucosa of the entire gastrointestinal tract, mainly in the lamina propria, but also in the smooth muscle layer of the intestinal wall. In humans, IMs in the lamina propria can be defined by its CD11c and interleukin-4-induced expression of 1 (IL-4I1). IMs in the human muscle layer can be distinguished from its lamina propria counterpart by the high expression of scavore receptors CD163 and COLEC12. In mice, IMs located in the laminae propria are marked by the expression of lymphocyte antigen 6c (Ly6C), CX3C chemokine receptor 1 (CX3CR1), F4/80, and CD121b (type II IL-1 receptor), and in addition to the expression of CX3CR1 and F4/80, IMs in the muscularis also have a high expression of CD206 (mannose receptor) and CD169 (sialic acid adhesin (Siglec) 1) [3,5]. Due to the difficulty in assessing IM function in the human context, most functional information has been gleaned from mouse studies. The blood mononuclear cells of mice expressed high levels of Ly6C and low levels of CX3CR1 [14,15]. With the entry of Ly6C^hi^CX3CR1^int^ monocytes into gastrointestinal tissues, under the influence of gut-specific signals (CSF1, TGF-β, IL-10, and CX3C chemokine ligand 1(CX3CL1)) and environmental factors (dietary-component-derived aryl hydrocarbon receptor (AhR) ligand and gut-microbiota-derived short-chain fatty acids (SCFAs)), monocytes undergo differentiation and gradually downregulated Ly6C expression, while increasing the surface expressions of F4/80, CX3CR1, and major histocompatibility complex II (MHCII) [16], thus becoming Ly6C^lo^F4/80^hi^CX3CR1^hi^MHCII^hi^ IMs (Figure 1).

IMs are involved in the recognition, phagocytosis, and degradation of cell debris and pathogens, and also play a role in presenting antigens to T cells, thereby initiating adaptive immune responses [17,18]. IMs are highly phagocytic and actively bactericidal, which, coupled with their location below the epidermal monolayer, means that they are ideally positioned to capture any material that disrupts the intestinal epithelial barrier [19]. In IBD patients, the phagocytosis and bactericidal effects of IMs are significantly reduced due to the damage, but the removal of bacteria can be achieved to a certain extent [20]. As secretory cells, IMs are critical for the regulation of immune responses and the development of inflammation, and they produce a variety of powerful chemicals, including enzymes, complement proteins, and regulatory factors. After digesting a pathogen, IMs will present the antigen of the pathogen to the corresponding Th cells. Presentation is accomplished by attaching antigen fragments to MHCII and integrating them into the cell membrane via exocytosis. Finally, antigen presentation leads to the production of antibodies that bind to the antigen of the pathogen. This makes it easier for IMs to attach to their cell membranes to realize phagocytosis [21]. Meanwhile, as an important participant in tissue repair, IMs can remove apoptotic cells and tissue debris, and produce anti-inflammatory as well as repair molecules for coordinated resolution [19]. During tissue injury, pathogens, infected cells, and necrotic cells release damage-associated molecular patterns (DAMPs) and pathogen-associated molecular patterns (PAMPs), which activate inflammatory signaling pathways in IMs, stimulate IM differentiation into IM2 phenotypes, and produce substances critical for restoring homeostasis, such as anti-inflammatory molecules (IL-10, TGF-β), growth factors (VEGF, PDGFA), and matrix metalloproteinases (MMP-8, MMP-10, and MMP-28), and actively promote tissue repair [22,23]. At the same time, IMs prevent excessive inflammatory responses to harmless commensal microorganisms and promote tolerance by producing IL-10.

According to the location of the IMs in the intestine, IMs can be divided into lamina propria macrophages (LPMs) and muscularis macrophages (MMs). LPMs are mostly differentiated from blood-derived monocytes [24,25]. LPMs are a population of cells very close to the intestinal wall, and the subepithelial localization of LPMs means that they are ideally positioned to capture and eliminate any bacteria that cross the epithelial barrier. Normally, they can secrete CSF1 to maintain survival, proliferation, and differentiation. When intestinal flora erode the intestinal epithelial cells, epithelial damage will be caused. At this time, LPMs will secrete IL-12 on T helper (Th) 1 cells, while secreting IL-1β and IL-23 on Th17 cells, in order to activate the immune response, reduce the inflammatory response, and repair the epithelial barrier [26]. LPMs also can maintain the integrity of the intestinal epithelial barrier by secreting factors that stimulate intestinal epithelial cell renewal, such as WNT ligands (WNTs) and prostaglandin E2 (PGE2) [27]. MMs are principally tissue-resident macrophages and located deeper in the intestine, relatively distant from the substances that pass through the intestine. MMs, together with the submucosal plexus, represent the internal innervation of the intestine, also known as the enteric nervous system (ENS). Bone morphogenetic protein (BMP) is a group of secreted proteins in the transforming growth factor β (TGF-β) family, which mainly controls organ development, and BMP receptor (BMPR) signaling is related to the differentiation of intestinal smooth muscle and intestinal neurons [28]. The BMP2 secreted by MMs forms the oligomerization of type I and II serine kinases of BMPR (BMPRIa and BMPRII) to transmit signals, which can activate BMPR, and the ligands bound to BMPR activate the classical mitogen-activated protein kinase (MAPK) signaling pathway through the phosphorylation and nuclear translocation of Smads 1, 5, and 8. Thus, it can change the contraction pattern of the intestinal smooth muscle, regulate intestinal peristalsis, and support the differentiation of intestinal neurons [29,30]. In turn, intestinal neurons promote the development and homeostasis of MMs by secreting colony-stimulating factor 1 (CSF1) (Figure 1).

IMs are divided into embryo-derived IMs (EIMs) and monocyte-derived IMs (MIMs) according to their origin. EIMs are derived from embryonic precursors and self-renew in situ, usually lasting throughout adulthood, and these IMs are long-term and locally maintained, coexisting with MIMs that are constantly replenished by monocytes [9,10]. EIMs are mainly located in the muscularis, while MIMs are mainly located in the lamina propria. EIMs can self-renew, which depends on growth factors such as M-CSF and GM-CSF [31]. Unlike MIMs, EIMs are terminally differentiated and cannot undergo a similar induction of polarization. EIMs exposed to IFN-γ and LPS (IM1 stimulation) or IL-4 (IM2 stimulation) under GM-CSF stimulation are similar to unpolarized IMs, and no release of cytokines, such as IL-10, is detected [32]. EIMs are inflammatory analeptic, characterized by the failure to produce inflammatory cytokines (IL-1, IL-6, IL-8, IL-10, IL-12, and TNF-α) and the downregulation of surface receptor expression (LPS, Fcα, Fcγ, CR3, and CR4 receptors) [16,33]. EIMs maintain homeostasis by performing IM2-like functions, where they efficiently remove bacteria and apoptotic cells without producing proinflammatory cytokines and antigen presentation [20,21]. The inflammatory anergy of EIMs, which is unique among all tissue macrophages, can promote the noninflammatory clearance of apoptotic and senescent cells by the cells and defend against microorganisms that may damage the mucosal epithelium.

**Figure 2:**
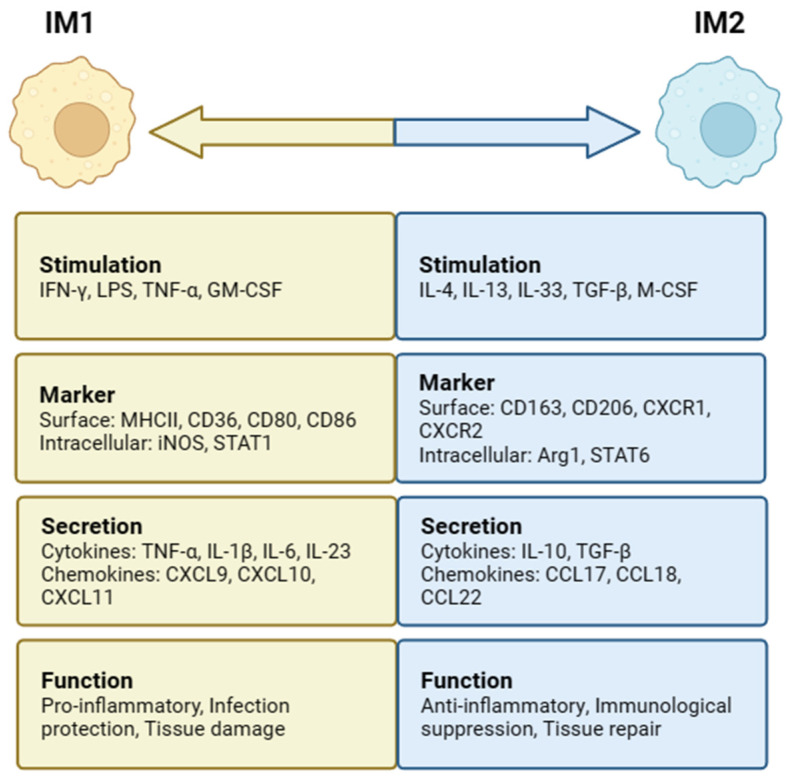
Intestinal macrophages (IMs) polarization and phenotypes. IM1 is stimulated by IFN-γ, LPS, TNF-α, and GM-CSF, while IM2 is stimulated by IL-4, IL-13, IL-33, TGF-β, and M-CSF. IM1 has functions such as proinflammation, infection protection, and tissue damage. IM2 has anti-inflammatory, immunological suppression, and tissue-repair functions.

In the intestine, MIMs can be induced to differentiate into two distinct subsets, IM1 and IM2, depending on the surrounding environmental stimuli (Figure 2). When IMs are stimulated with interferon-γ (IFN-γ), lipopolysaccharide (LPS), tumor necrosis factor-α (TNF-α), and granulocyte–macrophage colony-stimulating factor (GM-CSF), classical IM1 activation can be induced. High levels of proinflammatory cytokines (IL-1β, IL-6, IL-12, and IL-23), reactive oxygen species (ROS), and nitrogen intermediates (ROIs and RNIs) are produced [34,35]. IM1 mainly causes a Thl-type immune response, plays a role in the defense against bacterial infection, aggravates inflammatory reactions and leads to tissue damage, upregulates the expression of intracellular inhibitor of cytokine signaling protein 3 (SOCS3), and activates inducible nitric oxide synthase (iNOS) to produce NO from L-arginine [35]. Stimulating factors, such as IL-4, IL-13, IL-33, TGF-β, and macrophage colony-stimulating factor (M-CSF), are conducive to IM2 subpopulation differentiation and induce IM2 to express arginase 1 (Arg1) as well as secrete anti-inflammatory cytokines, including IL-10 and TGF-β [11]. IM2 mainly induces a Th2-type immune response and plays an important role in the defense against parasitic infection, promoting tissue repair and regulating immunological suppression [36].

## 3. Pathological Changes in IMs in IBD

The disturbance of homeostasis among immune cells, such as macrophages, epithelial cells, and microorganisms, in the intestinal environment is the main cause of IBD. IMs interact with various cells and microorganisms in the intestine, release different factors, change the inflammatory environment of the intestine, and cause the occurrence, development, or remission of IBD.

### 3.1. Effects of the Intestinal Environment on IMs

Intestinal epithelial cells (IECs) are adjacent to LPMs, and there is some interaction between the two cells. IECs are an important source of chemokines that attract monocytes in intestinal inflammation, and TGF-β as well as IL-8 from IECs can attract peripheral monocytes into the intestinal mucosa, and then differentiate into MIMs [37]. IECs express a wide range of pattern-recognition receptors (PRRs), such as the Toll-like receptor (TLR) and NOD-like receptor (NLR). Through these PRRs, MIMs can actively sense various bacterial stimuli and produce immune regulatory factors. For example, LPS (a TLR4 ligand)-stimulated MIMs are an important source of IL-10 and TGF-β in the intestinal mucosa, two important immunosuppressant cytokines responsible for inhibiting MIMs from producing inflammatory cytokines, suggesting that IECs can influence the anti-inflammatory effects of MIMs [38]. In addition, different IEC subgroups have different ways of regulating MIM functions. Goblet cells are a subgroup of IECs whose main function is to synthesize and secrete mucin as well as antimicrobial peptides (AMPs). The RELMβ of goblet-cell-specific AMPs can upregulate the expression of TNF-α, IL-12, IL-23, and MHCII in MIMs, as well as promote the establishment of Th1, leading to a proinflammatory immune response. Thus, intestinal inflammation caused by bacterial infection is aggravated [39]. Microfold cells (M cells) are a special type of IEC whose main function is to collect luminal antigens and transport them to the subepithelial lymphatic follicles. Studies have shown that M cells absorb enterohaemorrhagic Escherichia coli and then transfer the Escherichia coli to MIMs, resulting in increased bacterial survival, inducing MIM apoptosis, and ultimately leading to the release of Shiga toxins into the blood, aggravating intestinal inflammation [40]. In recent years, Tuft cells, a particular isotype of IEC, have been shown to regulate intestinal immunity [41]. Tuft cells, a major source of IL-25 in the gut, can regulate intestinal inflammation by promoting a Th2 immune response and affecting the anti-inflammatory function of MIMs.

After the proliferation and activation of intestinal fibroblasts are enhanced and activated, growth factors such as M-CSF and GM-CSF will be secreted to affect the differentiation and survival of IMs [25]. Another subgroup below the hematopoietic cell layer is intestinal fibroblasts, which tend to differentiate into IMs in response to environmental stimuli. Under the effect of M-CSF, IMs can differentiate into the IM2 type with an anti-inflammatory ability. Under the action of GM-CSF, they preferentially differentiate into IM1 with a proinflammatory ability. Myofibroblasts, as activated intestinal fibroblasts, can produce various IM regulatory factors (such as CCL2, IL-6, M-CSF, and TNF-α) in response to inflammatory stimuli. Studies have shown that myofibroblast-derived osteopontin (OPN) increases the IM2-type macrophage differentiation of IMs by binding to αvβ3 and CD44 [42].

Intestinal flora are a key mediator of IM differentiation and play an important role in the formation of IM phenotypes. On the one hand, they directly release short-chain fatty acids (SCFAs) and aromatic hydrocarbon receptor (AhR) ligands derived from dietary components; on the other hand, they indirectly induce IECs to release tolerance-inducing signals, such as TGF-β [43]. Intestinal flora catabolic primary bile acids turn into secondary bile acids in the intestine. These bile acids regulate immunity by reducing the expression of proinflammatory cytokines in monocytes, IMs, and dendritic cells. At the same time, they compete with pathogens for nutrients and produce SCFAs, which affect the growth of IECs and maintain intestinal homeostasis, effectively protecting the host from bacterial infection. Butyric acid is a kind of SCFA which is mainly secreted by Bacteroidetes and Firmicutes, and can downregulate the transcription of proinflammatory factors IL-6, IL-12, and iNOS in IMs [44]. Butyric acid also acts as a histone deacetylase inhibitor (HDACi), which can inhibit the inflammatory response by inhibiting the activation of NF-κB and producing proinflammatory mediators, restoring intestinal immune homeostasis, increasing oxidative phosphorylation, promoting the activation of IM2, inducing the antibacterial activity of IMs in vivo, and increasing drug resistance to intestinal pathogens [45].

### 3.2. Effects of IMs on the Intestinal Environment

At the same time, IMs can also affect the intestinal environment, thus affecting IBD. IMs maintain the integrity of the intestinal epithelial barrier by secreting factors that stimulate IEC renewal, such as WNTs and PGE2 [27]. Monocytes and IM1 macrophages promote the expression of claudin-2, a tight-junction protein, and the phosphorylation of a myosin light chain (MLC), which is associated with actin ring contraction and increased macromolecular permeability. In contrast, undifferentiated IMs and IM2-type macrophages inhibit the expression of claudin-2 and promote the expression of the bonding adhesion molecule JAMa, the MARVEL protein tricellulin, and cadherin E, thereby promoting the formation of tight junctions, constituting the paracellular channels of ions and molecules through IECs, which can selectively seal the paracellular space of IECs, control the passage of water and ions, reduce intestinal permeability, and relieve IBD [46,47]. This suggests that undifferentiated IMs and IM2-type macrophages promote intestinal epithelial barrier tightness, while monocytes and IM1 macrophages induce intestinal epithelial barrier permeability [48].

IMs play an important role in regulating the abnormal angiogenesis of intestinal inflammation. Upon sensing angiogenic signals, IMs migrate to the location of the neovasculum and secrete angiogenic cytokines, including NO and various proteases, to stimulate intestinal vascular endothelial cell proliferation or provide a favorable ecological niche for neovascular growth [49]. IM–intestinal vascular endothelial cell interaction is protective in IBD. IMs maintain intestinal homeostasis by preventing intestinal vascular endothelial leakage, and IM-derived HB-EGF preserves villus blood flow and microvascular structure, thus improving necrotizing colitis [50]. At the same time, MIMs actively participate in the remodeling and healing of damaged arteries and arterioles in the context of ischemic injury.

IMs play a key role in wound healing by secreting TGF-β, which can activate intestinal fibroblasts [42]. TGF-β is a powerful cytokine that promotes wound healing in the process of wound repair, and its abnormal expression directly affects the time of wound healing. TGF-β is also an important marker of regenerative epithelization, an essential condition for the formation of the skin matrix and granulation tissue, and can affect various stages of the healing process, providing a signal substance for normal healing. TGF-β secreted by IMs can promote the activation and proliferation of intestinal fibroblasts. In addition, IM-derived IL-36α can promote intestinal mucosal healing, in part by activating IL-36R signaling in intestinal fibroblasts, thus achieving the therapeutic effect of IBD. It is worth noting that the increase in the Wnt ligand of CD16^+^ IMs leads to abnormal accumulation of intestinal fibroblasts, which leads to the aggravation of intestinal fibrosis. This suggests that, while contributing to intestinal mucosal healing, the overactivation or accumulation of intestinal fibroblasts may lead to intestinal fibrosis, a nearly irreversible disease that can lead to permanent intestinal dysfunction in patients with IBD.

## 4. Therapeutic Strategies Targeting IMs for IBD

At present, the general principles of IBD treatment are as follows: to control acute attacks, relieve disease, reduce recurrence, and prevent complications. The general policy of drug treatment for IBD is a step-up therapy, and 5-aminosalicylic acids (5-ASAs), such as mesalazine, are preferred for mild IBD. For moderate IBD, glucocorticoids are the drug of choice, and immunosuppressants, such as azathioprine and methotrexate, are recommended for hormone resistance or dependence. For severe IBD, biologics with low side-effects but high costs, such as TNF inhibitors, could be selected. When patients have severe complications, such as intestinal obstruction, intestinal perforation, etc., surgery is required. Most classical IBD therapies affect IM function in a variety of ways, such as inhibiting inflammatory signaling pathways and inducing IM2-type macrophage differentiation. In the following sections, we provide a detailed summary of the current therapeutic strategies involving IMs and possible new options (Table 1).

### 4.1. Chemicals

Currently, 5-ASAs, glucocorticoids, and immunosuppressants are the main chemical drugs used to treat IBD. The 5-ASAs can directly inhibit the activation of the NF-κB signaling pathway, which is a major proinflammatory pathway in IMs, and can be detected in IBD patients’ inflammatory IMs. At present, the main oral drugs of 5-ASAs include mesalazine, oxazine, balsalazide, and mesalazine sulfapyridine. The main dosage forms of a 5-ASA preparation for external use include a suppository, an enema, and a foam preparation. The commonly used glucocorticoids are prednisone, hydrocortisone, methylprednisone, and budesonide. Corticosteroids (CSs) may be used in patients with mildly active IBD who do not respond to 5-ASAs, and CSs may also be used for the remission of moderate to severe IBD. CSs bind to specific cytoplasmic receptors to inactivate the proinflammatory transcription factor NF-κB and nuclear transcription factor activating protein-1 (AP-1), while promoting the differentiation of monocytes into alternately activated IM2-type macrophages, increasing anti-inflammatory typing and alleviating intestinal inflammation [50,51].

Common immunosuppressants include azathioprine, 6-mercaptopurine, and methotrexate. Methotrexate may be considered in children with CD who are intolerant to purines or find them ineffective. Methotrexate inhibits IM proinflammatory gene expression by reducing the expression of thymidine kinase synthetase [26], suggesting that immunosuppressants can improve IBD by regulating IMs.

### 4.2. Biologics

In the process of IBD, an increase in the TNF-α level could increase the apoptosis of intestinal epithelial cells and promote the infiltration as well as the activation of immune cells, such as IMs, thus causing intestinal damage. Anti-TNF-α monoclonal antibodies currently approved by the FDA for the treatment of IBD include infliximab, adalimumab, golimumab, and certolizumab pegol. Anti-TNF-α monoclonal antibodies can isolate TNF-α and prevent the TNF-α receptor (TNF-αR)-mediated inflammatory activation of IMs. Studies have shown that the immunosuppressive properties of anti-TNF-α monoclonal antibodies depend on their Fc region, which induces the differentiation of anti-inflammatory CD206^+^ IM2 by binding to the FCγ receptor (FCγR) in order to block the intestinal inflammatory response mediated by TNF-α and TNF-αR, and play a major role in wound healing [52,53]. Under inflammatory stimulation, the FCγR junction rapidly activates ERK, leading to histone modification at the IL-10 promoter and inducing the effective transcription of IL-10. ERK activation must be combined with inflammatory stimulation to induce IL-10 [56]. Thus, anti-TNF-α monoclonal antibodies increase IL-10 secretion in IMs in an FCγR-dependent manner. However, biologics without Fc fragments, such as certolizumab pegol, cannot induce anti-inflammatory IM2 differentiation [55]. This could be used to consider the development of new antibody-based therapies that could be an entirely new avenue of treatment for IBD. In spite of the positive therapeutic effects of anti-TNF-α monoclonal antibodies, the nonselective blocking of TNF-α in the system also brought about significant adverse reactions, such as immune-related infections and the generation of antidrug antibodies. Therefore, anti-TNF-α therapy for IBD was effective but needed to be limited to specific inflammatory sites.

Studies have shown that the intervention of intestinal vascular endothelial cell adhesion is effective in the clinical treatment of IBD [51]. Vedolizumab, an integrin receptor antagonist, is a humanized IgG1 monoclonal antibody that specifically targets α4β7 integrin, inhibits binding to the intestinal vascular endothelial cell surface ligand MAdCAM-1, and prevents infiltration of α4β7-expressing T cells into the inflamed intestine. Vedolizumab significantly reduced the number of IM1 macrophages in the gut of IBD patients, while increasing the number of IM2-type macrophages, which alleviates intestinal inflammation, and is suitable for moderate to severe UC and CD. The proinflammatory cytokines TNF-α and IL-1β can induce MAdCA IM1 expression in intestinal vascular endothelial cells, and the NF-κB and PI3K/Akt signaling pathways are also necessary for this process [55].

### 4.3. Other Possible Therapeutic Strategies

In addition to cytokine-based therapies, bacterial metabolites can also be used to relieve IBD. Butyric acid is a product of microbial fermentation, which is metabolized mainly in IECs, and can increase the expression of antimicrobial peptides (AMPs) and tight-junction (TJ) proteins, helping to maintain the intestinal epithelial barrier [55]. This short-chain fatty acid can also inhibit the activation of the IM1 and promote the differentiation of IM2, thus alleviating intestinal inflammation.

In IM-related therapy for IBD, the cell-specific response of a signaling-pathway-based approach may make therapeutic outcomes unpredictable. For example, many pathogenic cytokines (IL-13, IL-23, and IFN-γ) are transmitted through the Janus kinase and transcriptional signal converter and activator (JAK/STAT) signaling pathway, so JAK inhibitors (tofacitinib) can be used clinically to treat IBD. However, JAK inhibitors also block several anti-inflammatory protective pathways, such as the IL-10/STAT3, IL-22/STAT3, and IL-4/STAT4 pathways [57]. Another example is the NF-κB signaling pathway, which is the major proinflammatory pathway in IMs and is considered a potential therapeutic target, but it can also promote the survival and proliferation of damaged IECs, thereby complicating the development of IBD-related therapies.

## 5. Conclusions

In recent years, the field of intestinal macrophage (IM) biology has made significant progress in the role of heterogeneity in determining the phenotypes and functions of IMs. The emergence of new technologies, particularly single-cell RNA sequencing (scRNA-seq), has revealed the true extent of heterogeneity. In humans and mice, there is a large degree of diversity between macro/microenvironments in different tissues and between different microenvironments in the same tissues, even without inflammation or infection. In vivo, common tissue macrophages, such as liver Kupfer cells, epidermal Langerhans cells, and central nervous system microglia, are derived from embryonic precursors and sustain themselves through in situ self-renewal throughout life. Unlike these tissue macrophages, IMs are initially derived from embryo-derived macrophages, but these are gradually replaced after birth by blood-monocyte-derived macrophages and are replenished throughout adulthood. IMs perform various functions in IM1 (proinflammatory) or IM2 (anti-inflammatory) phenotypes to affect a wide range of diseases, such as infectious diseases, inflammatory diseases, and malignancies.

Intestinal bacteria and other components in the intestine, such as fungi and viruses, can cause ecological dysregulation and IM activity dysregulation in IBD by interacting with diet. To study this relationship, developing mouse models with intestinal microbiomes that are consistent with the complexity and diversity of humans can help. The dysregulation of IM activity can lead to excessive tissue repair and the development of intestinal fibrosis. However, the involvement of IMs in intestinal fibrosis is rarely understood at present, and the further study of it is necessary.

Moreover, the role of IMs in supporting and maintaining the enteric nervous system (ENS) opens the door to further research on how to harness the therapeutic potential of these cells in the context of gastrointestinal neurodegenerative diseases and provides new directions for the development of new therapies in the future.

## Figures and Tables

**Table 1:** Therapeutic strategies targeting IMs for IBD.

	Name	Therapeutic Mechanism	References
Chemical	5-ASAs	Inhibit the activation of the NF-κB signaling pathway	[50]
	Glucocorticoid	Inactivate NF-κB and AP-1 and promote the differentiation of IM2-type macrophages	[51]
	Immunosuppressant	IM proinflammatory gene expression was inhibited by reducing the expression of thymidine kinase synthetase	[52]
Biologic	Anti-TNF-α monoclonal antibody	The Fc region binds to FCγR, inducing anti-inflammatory IM2 phenotype differentiation and increasing IL-10 secretion in IMs	[53,54]
	Integrin receptor antagonist	The intervention of intestinal vascular endothelial cell adhesion	[55]

## Data Availability

No new data were created or analyzed in this study. Data sharing is not applicable to this article.
